# A Bio-Inspired Herbal Tea Flavour Assessment Technique

**DOI:** 10.3390/s140712233

**Published:** 2014-07-09

**Authors:** Nur Zawatil Isqi Zakaria, Maz Jamilah Masnan, Ammar Zakaria, Ali Yeon Md Shakaff

**Affiliations:** 1 Centre of Excellence for Advanced Sensor Technology (CEASTech), Universiti Malaysia Perlis, Jejawi 02600, Perlis, Malaysia; E-Mails: mazjamilah@unimap.edu.my (M.J.M.); ammarzakaria@unimap.edu.my (A.Z.); aliyeon@unimap.edu.my (A.Y.M.S.); 2 School of Mechatronic Engineering, Universiti Malaysia Perlis, Pauh Putra Campus, Arau 02600, Perlis, Malaysia; 3 Institute of Engineering Mathematics, Universiti Malaysia Perlis, Pauh Putra Campus, Arau 02600, Perlis, Malaysia

**Keywords:** bio-inspired flavour assessment, e-nose, e-tongue, low level data fusion, intermediate level data fusion, classification, LDA, SVM, KNN, PNN, GC-MS

## Abstract

Herbal-based products are becoming a widespread production trend among manufacturers for the domestic and international markets. As the production increases to meet the market demand, it is very crucial for the manufacturer to ensure that their products have met specific criteria and fulfil the intended quality determined by the quality controller. One famous herbal-based product is herbal tea. This paper investigates bio-inspired flavour assessments in a data fusion framework involving an e-nose and e-tongue. The objectives are to attain good classification of different types and brands of herbal tea, classification of different flavour masking effects and finally classification of different concentrations of herbal tea. Two data fusion levels were employed in this research, low level data fusion and intermediate level data fusion. Four classification approaches; LDA, SVM, KNN and PNN were examined in search of the best classifier to achieve the research objectives. In order to evaluate the classifiers' performance, an error estimator based on k-fold cross validation and leave-one-out were applied. Classification based on GC-MS TIC data was also included as a comparison to the classification performance using fusion approaches. Generally, KNN outperformed the other classification techniques for the three flavour assessments in the low level data fusion and intermediate level data fusion. However, the classification results based on GC-MS TIC data are varied.

## 1. Introduction

The number of food and beverage industries is growing due to an increase in national and international demand. The business opportunity attracts the herbal industry to penetrate the herbal-based products market. Since herbal-based products are considered to have medicinal value [1], strict regulations are applied for such products to enter the market. Moreover production of herbals in the form of herbal medicines intended for preventive or therapeutic use would be regulated as drugs under the federal laws. Thus, simply by producing herbal-based product as foods, dietary supplements and health drinks which seems to be more beneficial, companies do not need to abide by these strict regulations anymore and the health claims on their labels are no longer an obligation of proof. One such example is the growing demand for herbal teas such as java tea made from *Orthosiphon stamineus* (*O. stamineus*) that is becoming more favourable among customers.

In product commercialization, especially foods and beverages, good quality products play an important role in order to boost sales. The quality of foods and beverages is characterized by several attributes. One of the most well-known attributes is the sensory quality. Sensory quality consists of sensory characteristics such as appearance, flavour (olfactory and taste sensations), texture and auditory properties. Thus, any of those characteristics like appearance for example, that seems to play only a single role is still considered important since it has forms part of the whole collection of food quality parameters [2]. However, the evaluation of characteristics differs according to the types of foods and beverages. For herbal teas, appearance is not as important since most herbal teas have almost identical amber colours. Therefore, taste is considered equivalent to an overall impression of flavour, since it is the most significant product feature convincing consumers about the authenticity of the herbs.

Instead of sustaining the original flavour, the existence of unpleasant tastes such as bitter tastes usually detected by kids, become another challenge for the food and beverage industries [3]. Thus, to overcome this problem, flavour masking techniques are applied to hide the unacceptable taste [4]. The concentration level of a herbal tea is also important, especially for herbs which have astringent activity. An astringent herb has an impact on the digestive, urinary and circulatory systems. However, it can be considered as toxic if taken in large amounts [5]. The concentration of a herbal tea is not only affected by the amount of leaves added to the water but also the duration of brewing time. The longer the brewing time, the more the bioactive ingredient(s) in the herb are extracted. Although the level of colour transparency of a herbal drink is usually applied as an indicator of tea concentration, the result of that indicator becomes inaccurate if products are prepared and placed in a dark tea cup. Alternatively, flavour assessment becomes an available and easy way to evaluate the concentration.

These varieties of flavours are usually assessed by human panels or gas chromatography-mass spectroscopy (GC-MS), which is time consuming, expensive and can only be done by panel experts. In addition, panel experts are able to evaluate but hardly quantify the flavour concentration of liquid objects. Thus, the need for bio-inspired flavour assessment methods that can imitate the biological functions of human flavour assessment is of interest nowadays. The advancement of currents technologies and innovation such as the electronic nose (e-nose) and electronic tongue (e-tongue) are preferred to replace the panel experts since both conventional and current approaches are believed to complement each other [6,7]. The fusion of these sensors is currently an ideal mechanism towards bio-inspired flavour assessment. They are inspired by the human flavour detection mechanism that mimics the human nose and tongue [8]. Basically, flavour is derived through the combination of the sense of taste and smell [9] together with the tactile sensations of the sense of touch [10]. Earlier, Wide *et al.* [11] noted that the combination of these sensors has the potential to mimic the human flavour panels since measurement data from the sensors are manipulated to produce sensor-specific opinions about the human-like sensing modalities. Later, Cole *et al.* [9] have successfully confirmed that flavour can be assessed by combining these two artificial sensors. Though the e-noses and e-tongues are not integrated since each device works with its own software package, data fusion techniques can be applied for further data analysis [12].

This paper discusses two types of data fusion approaches, namely low level data fusion (LLDF) and intermediate level data fusion (ILDF). These sensors are analysed by further enhancement of its capability to classify simple and complex flavours by using pattern recognition system derived from array sensors. Based on recently reported works, herbal flavour assessment can be achieved through different classification techniques, including Linear Discriminant Analysis (LDA), *k*-Nearest Neighbour (KNN), Support Vector Machine (SVM) and Probabilistic Neural Network (PNN) [13,14]. In this research, three flavour conditions of herbal tea will be investigated by using the proposed technique: (1) different types and brands of herbal tea; (2) different concentrations of herbal tea and (3) different masking agents.

## 2. Materials and Methods

### 2.1. Sample

#### 2.1.1. Selection

For the purpose of this experiment, seven different types of tea were involved. Of these, there are four different herbal tea brands of commercial *Orthosiphon stamineus* (labelled as HPA, RH, PH and POL according to the manufacturer names), a fresh coarsely ground *O. stamineus* dried leaves sample (Agro) obtained from our home-grown plants (UniMAP's Sungai Chuchuh plantation), and two different types of commercial *Camelia sinesis i.e.*, green tea (GT) and black tea (BT) bought from the nearest local store. In order to ensure no bias influencing the storage effect, all teas were removed from the tea bags and stored in stainless steel canisters. For the preparation, the same brewing method is applied for all different tea types. For each tea type, seven different tea infusions are prepared and five repetitions of the measurements are recorded for further classification. In addition, three different bitter taste masking agents such as honey (H), sugar cane (SC), and strawberry (SB) are applied for the flavour masking investigation.

#### 2.2.2. Preparation

Herbal drink equivalent to 2 g of each brand and also 1, 2, 3 and 4 g from the home-grown plants (labelled as Agro) were infused with 200 mL boiled distilled water for 5 min and filtered using a stainless steel sieve for tea preparation. The filtrate is immediately cooled to about 25 °C under tap water. In addition, 2 mL of masking agent was added into the tea infusion (with 2 g Agro sample). This solution was be stirred using a magnetic stirrer set at about 800 rpm to ensure a homogenous solution.

**Figure 1.**
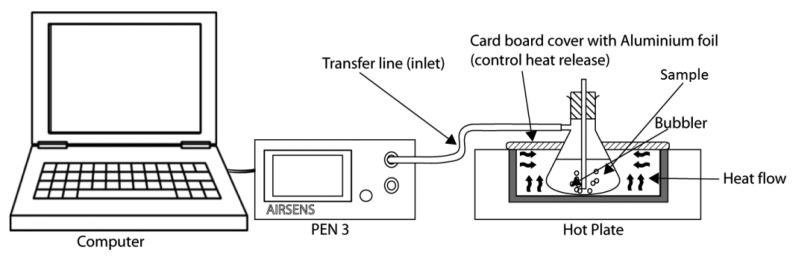
E-nose setup for volatile compound evaluation of teas of different type, concentration and bitter masking agent samples.

**Figure 2.**
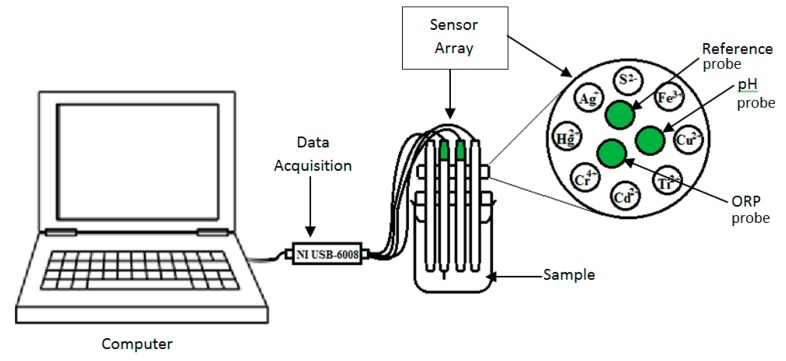
E-tongue setup for non-volatile compound evaluation of teas of different type, concentration and bitter masking agent samples.

Then 5 mL of the tea infusion was placed into 20 mL vials and the rest (195 mL) was placed into a filtering flask and covered with a silicon stopper as illustrated in Figure 1. The chamber is covered with Parafilm™ to avoid any volatile compounds from exiting the flask. To stabilize the concentration of tea aroma in the headspace of the filtering flask, the flask with tea infusion was kept for 10 min at 35 °C using a hot plate in a dark (wrapping the flask with aluminium foil) and then further analysed by the e-nose. The same prepared sample was used for the e-tongue as shown in Figure 2.

### 2.2. E-Nose and Measuring Condition

Experiments were performed using PEN3, WMA (Win Muster Airsense) Analytics Inc., Schwerin, Germany. PEN3 comprises a sampling tool, a chamber consists of an array of sensors, and pattern recognition software (Win Muster v.1.6.2.14) for data logging. The sensor array was making up of 10 MOSs (Table 1). The sensor response was indicated as the ratio of conductance (G/G_0_).

During the measurement process the headspace gas was pumped into the sensor chamber with a constant rate of 400 mL/min via Teflon tubing connected to a needle. When the gas accumulated in the headspace of the vials and was pumped into the sensor chamber, the ratio of conductance of each sensor changed. After complete detection of each sample, clean air (filtered through activated charcoal) was used to wash the sensor for a period of time and to allow the sensor to recover to a stable state.

The measurement procedure was controlled by the Win Muster v.1.6.2.14 computer program. The measurement phase lasted for 30 s, enough for the sensors to reach a stable status. The interval for data collection is 0.2 s. A computer recorded the responses of the e-nose every 0.2 s. When the measurement was completed, the acquired data was properly stored for later use. Then, the chamber is cleaned using activated carbon filtered air for 85 s. The temperature of the filtering flask was kept at 35 ± 1 °C using a hotplate. The filtering flask is covered with aluminium foil to avoid temperature gradient effects.

### 2.3. E-Tongue and Measuring Condition

Experiments were performed using chalcogenide-based potentiometric sensors with eight distinct ion-selective sensors from SENSOR SYSTEM, LLC (St. Petersburg, FL, USA) [12]. This potentiometric sensor is designed to be partially selective. The e-tongue sensors were developed by arranging eight of the potentiometric sensor around a reference, pH and ORP probe. Each sensor output was connected to two analogue inputs of a data acquisition board (NI USB-6008) from National Instruments (Austin, TX, USA) and the reference probe is connected to the common ground of the board.

The sensor array was dipped for 2 min in distilled water (stirred at 800 rpm) at the beginning of the experiment. After each sampling, the sensor array was rinsed twice using distilled water (stirred at 400 rpm for 2 min) again to remove any residues from previous sample sticking on the e-tongue which could contaminate the next sample. In each measurement, the sensor array was steeped simultaneously (sensor tip 2 cm below the solution level) and left for 5 min, and the potential readings were recorded for the whole duration.

### 2.4. SPME and GC-MS Analysis Setting

Solid-phase microextraction (SPME) needles made by CAR/PDMS (Supelco-57320-U, Bellefonte, PA, USA) were used to extract the headspace of aroma released from the solution. The SPME was used as sample introduction method and mass spectrometry (MS) as a detector for gas chromatography (GC). About 5 mL of herbal tea extract was used for this purpose.

#### 2.4.1. SPME-GC Setting

An SPME fiber (75 lm Carboxen-PDMS; Supelco, Inc., Bellefonte, PA, USA) was exposed to the sample headspace in a 20 mL vial heated with a hotplate at 80 °C for 10 min. The Volatile Flavour Compounds (VFCs) were desorbed by inserting the SPME fibre into a GC injector (injector temperature 230 °C) in split less mode connected with a fused-silica GC column (Elite 5MS, 30 m, 0.25 mm ID, 0.25 μm film thickness) (Perkin Elmer, Waltham, MA, USA) for 15 min. The initial temperature of the GC was set at 40 °C for 4 min, and then the oven temperature was increased at a rate of 5 °C/min until it reaches 230 °C where it remained for another 3 min. The detector temperature was set at 250 °C.

#### 2.4.2. GC-MS Setting

For GC-MS analysis, a GC (Clarus 680) coupled with a mass spectrometer (Clarus 600T, Perkin Elmer) was used. The GC operating conditions (temperature and time) were the same as described above. The mass spectrometer was operated in the electron-ionization (EI) mode at an ionization voltage of 70 eV. To support the findings of this research, the GC-MS output was also analysed using LDA, KNN, SVM and PNN. Usually GC-MS provides information on specific analytes of interest (selective ion monitoring (SIM)) and mass spectra data (SIM and total ion current (TIC)). In this research, TIC data was used as input for the classifier. TIC is merely the sum at each time point of every *m/z* value across a mass spectrum [15].

### 2.5. Data Division

**Figure 3.**
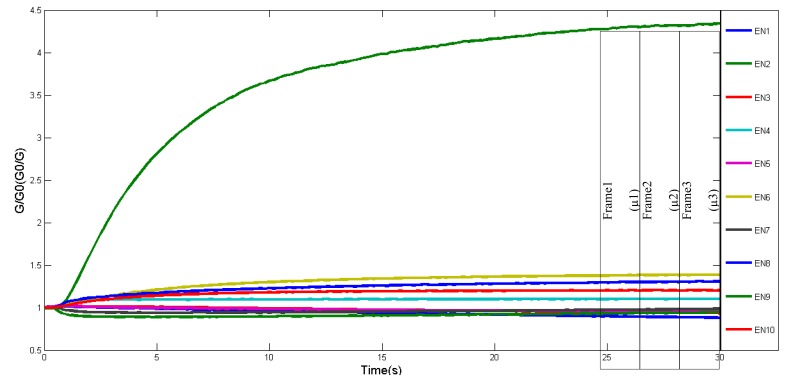
E-nose characteristic response curve of 10 array sensor values during tea infusion sample measurement.

**Figure 4.**
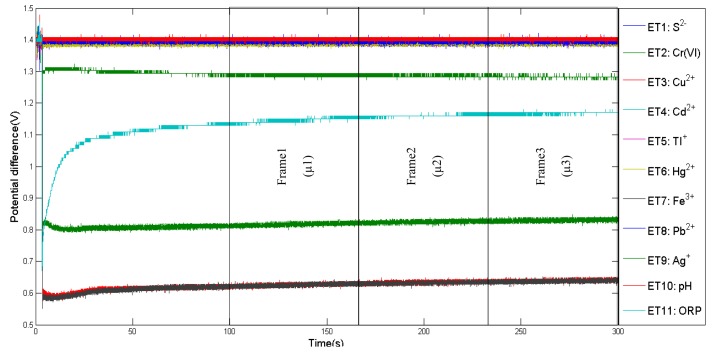
E-tongue characteristic response curve of 11 array sensor values during tea infusion sample measurement.

In order to obtain reliable data, the optimum time frame for data collection is determined. For instance, [16] have proposed stable phase values as responsive values for data collection. For this experiment, response curves of 10 applied sensors during tea infusion sample measurements are shown in Figure 3. Initially, the conductivity of each sensor was low, and then increased and stabilised after 25 s. Between 25 s to 30 s, the data was divided into three partitions. Two out of three frames will be assigned randomly for training and the remaining frame for testing. In addition, the mean for each frame was calculated and represented one observation value. The same approach was applied for e-tongue data as shown in Figure 4, where selected points begun at 100 s and ended at 300 s. The number of observations used for training and testing are listed in Table 2.

### 2.6. Pattern Recognition of E-Nose and E-Tongue Data

Before any classification process was done, data from each modality were fused using two approaches, namely low level data fusion (LLDF) and intermediate level data fusion (ILDF). In LLDF, raw data from the e-nose and e-tongue are combined before any further classification process, whereas in ILDF extracted significant features from the e-nose and e-tongue are combined before any further classification process. For the purpose of ILDF, LDA was applied in extracting important features to reduce the data dimensionality. The number of discriminant functions selected for further classification is determined by the number of class and feature. Details of each fusion level are referred to in [17].

For the data analysis, four types of classification techniques—LDA, KNN, PNN and SVM were examined. In order to evaluate the classifiers' performance, an error estimator based on a resampling method such as cross validation was applied during classifier parameter optimization. For KNN, SVM and PNN classifier, a k-fold cross validation technique was applied using k = 10, and the analyses were performed using MATLAB R2013, whereas for LDA, leave-one-out cross validation was employed for the error estimation and the analyses were performed using SPSS17.

LDA is a well-known classical statistical technique that finds the projection that maximizes the ratio of scatter among data of difference classes to scatter within data of the same class [18]. For the purposes of this research, the Fisher criterion was applied in order to provide the highest possible discrimination between different classes of data to help us to classify the data accurately. In addition, the Fisher criterion does not require data to follow a Gaussian distribution. PNN is a part of a radial basis network that is implemented based on a predominant nearest neighbour classifier. The classification factor is highly dependent on the spread of its radial basis function [14]. The spread values for each dataset were determined based on the lowest mean square error (MSE) value during parameter optimization stage, that is, during the training stage. These values are outlined in Table 3.

KNN is the simplest method used to decide the class to which a sample belongs to and it is a popular nonparametric method. Number of neighbours (*k*) and type of distance are important parameters in developing KNN models. Values for each parameter used in this research for each dataset are listed in Table 4. These values were chosen based on the lowest MSE. SVM is known as a current method to classify gene expression data. It works by separating space into two regions by a straight line or hyper plane in higher dimensions. Generally, the patterns are not linearly separable, therefore first they were mapped into a high dimensional space using a proper kernel, and then the optimization was carried out. For mapping the data into high dimensional space, a radial basis function (RBF) kernel transformation was used for the first and second dataset while a linear kernel was used for the third dataset. In the current study, Least Square SVM (LS-SVM) was used for the first and second dataset, while Sequential Minimal Optimisation SVM (SMO-SVM) was applied for the third dataset in finding the separating hyper plane. All these were set based on the lowest MSE during parameter optimisation. Details on the gamma value for the RBF kernel and the box constraint are listed in Table 5. For further reading about the KNN and SVM methods readers are referred to [19].

Evaluation for each classifier is determined through performance measures such as positive predicted value (PPV) and accuracy is calculated on both stage data (training and testing); sensitivity and specificity are calculated on testing data only. Since this research involve multiple classes, the sensitivity and specificity of classifier on each class was also counted. In this research, sensitivity is the probability that a classifier will produce a positive result on a respective class population. Specificity is the probability that a classifier will produce a negative result when used on another class population. Accuracy is the ratio of the number of correctly classified samples to the total number of samples. PPV is the proportion of samples with positive result that are correctly classified. For further information on selectivity, specificity and accuracy readers may refer to [13].

## 3. Results and Discussion

Data analyses were performed using MATLAB R2013 and SPSS17. Findings of the analyses are described based on different fusion levels for each studied dataset.

### 3.1. Features Extraction

#### 3.1.1. E-Nose

For the different types of tea and manufacturer dataset, seven groups (*G* = 7) are involved with 10 features (*p* = 10) for the e-nose. The number of useful discriminant function that can separate the tea sample by types and brands is the minimum of (*G* – 1) or *p*, which in this case it is the minimum of six (*G* – 1) or 10 (*p*), that is six. Thus, there will be six useful discriminant functions that can be applied to separate the tea flavours by type and manufacturer using the 10 gas sensors' features.

Table 6 illustrates the list of standardized LDA coefficient for each discriminant function. There are about six discriminant functions comprised for all the features, *EN*1 to *EN*10. All discriminant functions are a linear combination of the features and it can be written in an equation such as Equation (1). The rest of the discriminant functions follow accordingly:

(1) $$\begin{array}{c} df_{\text{EN}1}=2.240\text{EN}1-1.153\text{EN}2+2.823\text{EN}3+0.580\text{EN}4-3.991\text{EN}5-0.884\text{EN}6\\ -1.539\text{EN}7-0.327\text{EN}8+2.915\text{EN}9+1.797\text{EN}10 \end{array}$$

where *EN*1 until *EN*10 are the conductance of the 10 gas sensors found in the tea samples. Discriminant scores for each observation were obtained by substituting the value from each feature into the discriminant function. These scores will be used as input features for further classification techniques.

The summary table for the total number of discriminant scores use for the ILDF classification stage for all datasets in this research is shown in Table 7.

#### 3.1.2. E-Tongue

For different types of tea and manufacturer dataset, seven groups (*G* = 7) are involved with 11 features (*p* = *11*) for the e-tongue. The number of useful discriminant functions that can separate the tea sample by types and brands is the minimum of (*G* – 1) or *p*, and in this case it is the minimum of six and 11, that is six. Thus, a maximum of six useful discriminant functions can be applied to separate the tea by type and manufacturer using the 11 potential metric electrode features.

Table 8 illustrates the list of standardized LDA coefficient for each discriminant function. All discriminant functions are a linear combination of the features (*ET*s) and it can be written in an Equation such as Equation (2). The rest of the discriminant functions are as follows:

(2) $$\begin{array}{c} df_{\text{ET}1}=5.657\text{ET}1+2.063\text{ET}2-0.182\text{ET}3+0.165\text{ET}4-0.099\text{ET}6-0.478\text{ET}7\\ +0.160\text{ET}8-4.656\text{ET}9-0.113\text{ET}11 \end{array}$$

where *ET*1 until *ET*11 are the potential difference found in the tea samples by the nine ion-selective electrode sensors. Features *ET*5 and *ET*10 are not included in this linear combination since those features failed in the tolerance test. Tolerance test is a test that used to identify useful features for better classification performance. Discriminant scores for each observation were obtained by substituting value from each feature into the discriminant function. These scores will be used as input features for further classification techniques. A summary of the total number of discriminant scores used for the ILDF classification stage for all datasets in this research is presented in Table 9.

### 3.2. Low Level Data Fusion (LLDF)

Once data were fused at the low level where data from e-nose and e-tongue are concatenated, samples were classified using different classification techniques. Table 10 shows the classification performance in terms of accuracy for the four techniques applied. For LLDF of the different types of tea and brands for the *O. stamineus* dataset, LDA and KNN outperformed SVM and PNN with 100% correct classification. The performance of SVM deteriorates when the test set was applied. This is due to the confusion in classifying the Agro tea compared to GT and BT tea. Accuracy of PNN was affected by the misclassification of PH and HPA tea brands. This may be due to the similarity of VOCs and/or chemicals shared by both brands that leads to the confusion. The performance of sensitivity and specificity for each classifier is shown in Table 11. LDA and KNN have a very good specificity and sensitivity in classifying the tea samples based on their types and brands. SVM in contrast, has low specificity since the classifier failed to detect the control sample (*i.e.*, Agro tea).

Classification performance for different concentrations dataset evaluated for different classifiers are tabulated in Table 12. It can be seen from the table that KNN provides the best classification with the lowest error rate, followed by SVM, PNN and LDA. It seems that for samples with high concentrations, all the classifiers performed well as compared to the low concentrations. In other words, the examined classifiers can correctly classify tea samples into the exact group when the concentration is high enough to differentiate the Agro tea. From the perspective of sensitivity and specificity, generally we may conclude that KNN, SVM and PNN can classify the correct Agro tea at higher concentration, while for other concentrations, the specificity of every classifier varies. Details of the sensitivity and specificity for different concentrations for Agro tea dataset in LLDF model are outlined in Table 13.

Table 14 shows the classification performance of different types of masking agent such as H, SB, and SC that were added into the Agro tea solution. All the classifiers performed well during the training, but PNN proved to be the best classifier in discriminating the solution of Agro tea when the test set is applied. From the perspective of specificity, all the classifiers are good in specifying the solutions of Agro tea with different masking agents into their actual group. However, the classifiers' sensitivity is uneven where LDA and KNN are concerned, and SVM was unable to locate the Agro tea solution with particular masking agents into the correct groups. Please refer to Table 15 for further details.

### 3.3. Intermediate Level of Data Fusion (ILDF)

In Table 16, all the classifiers recorded no errors during training, but KNN outperformed other classifiers during testing without errors followed by LDA with 0.4% error caused by misclassifying the PH brand.

It seems that SVM and PNN are unable to allocate the Agro tea sample to its correct group. According to the sensitivity and specificity data shown in Table 17, again KNN proves to be the best classifier that fulfils the sensitivity and specificity demands by allocating the correct tea samples of different type and brand.

Overall, for the classification of tea sample according to different concentrations, the performance of all the classifiers was varied and lower compared to the different types and brands dataset. Although KNN and PNN performed well during training, KNN outperformed PNN, LDA and SVM with a 5.7% error in misclassifying tea samples that contained 1 g and 2 g *O. stamineus*. Notice that all the classifiers are able to allocate correctly tea samples for the higher concentration of 4 g *O. stamineus*. See Table 18 for further details. In addition, all classifiers have high specificity for the sample with 4 g *O. stamineus* as presented in Table 19. Compared to LLDF, all classifiers performed better in the LLDF framework.

In classifying different types of masking agent dataset, KNN and SVM outperformed LDA and PNN with 100% accuracy when tested using new data as illustrated in Table 20. As for the sensitivity and specificity in Table 21, the performance of KNN and SVM are consistent for both criteria in detecting different types of masking agent as well as in allocating the solutions of Agro with different masking agents. Although the performance of LDA is better than that PNN, this classifier has low specificity on the Agro product, as 2.9% of the Agro product was assigned as Agro + SB which affected the LDA sensitivity on Agro + SB. Compared to the LLDF framework, KNN and SVM performed better in ILDF while for PNN is was the other way around.

### 3.4. Gas Chromatography Mass Spectrometry (GC-MS)

Findings for the classification of tea using GC-MS based on types and brands are illustrated in Table 22. All the applied classification approaches performed well in the training. However, only KNN provided perfect accuracy in allocating the GC-MS TIC of tea based on types and brands correctly, followed by SVM with 97% of accuracy where the error was caused by the GC-MS TIC misclassifying the black tea. Table 23 displays the sensitivity and specificity of each classifier. From the result, it can be seen all the classifiers are inconsistent in terms of their sensitivity in detecting the correct groups for GC-MS TIC of tea types and brands. Conversely, almost all the classifiers have higher specificity in classifying the GC-MS TIC of tea types and brand, except for brand PH where LDA and PNN were unable to classify it correctly. It seems that for BT type, the specificity of all the classifiers also fails to detect it correctly in the GC-MS TIC of the tea types and brands.

For the different concentrations dataset, distilled water is not included in this analysis due to the GC-MS limitation as shown in Table 24. All proposed classifiers have 100% accuracy during the training stage and become worse during the testing stage. Only 60% accuracy was achieved by LDA and SVM in classifying different concentrations of the *O. stamineus* GC-MS TIC dataset. PNN had the lowest performance during testing, where only 25% accuracy was achieved. Besides, this classifier is not selective on the sample with 1 g *O. stamineus* as shown in Table 25. It can be observed that the classification performance using LDA, KNN, SVM and PNN based on the GC-MS output is not as good as the results of the fusion method.

### 3.5. Proposed Automatic Bio-Inspired Flavour Assessment and Grading System

**Figure 5.**
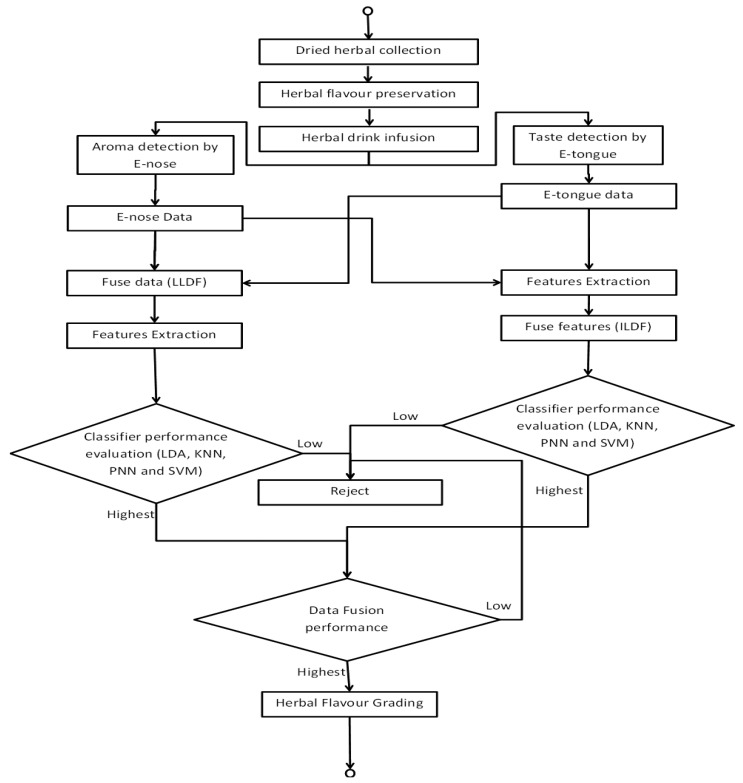
Bio-inspired Flavour assessment and grading system via data fusion.

Once the best classifier and data fusion level suitable for the flavour assessment were identified, an automatic bio-inspired flavour assessment and grading system is proposed. The whole process of the automatic flavour assessment and grading system is described in Figure 5. It is a complete process necessary in implementing flavour assessment and grading that is derived from several different procedures. Basically flavour of a new sample set that are to be assessed and graded is based on the calculated distance applied in the KNN classifier. The advantage of the proposed system is that it can provide specific flavour grading of the test samples that will be visible in the classification result.

**Figure 6.**
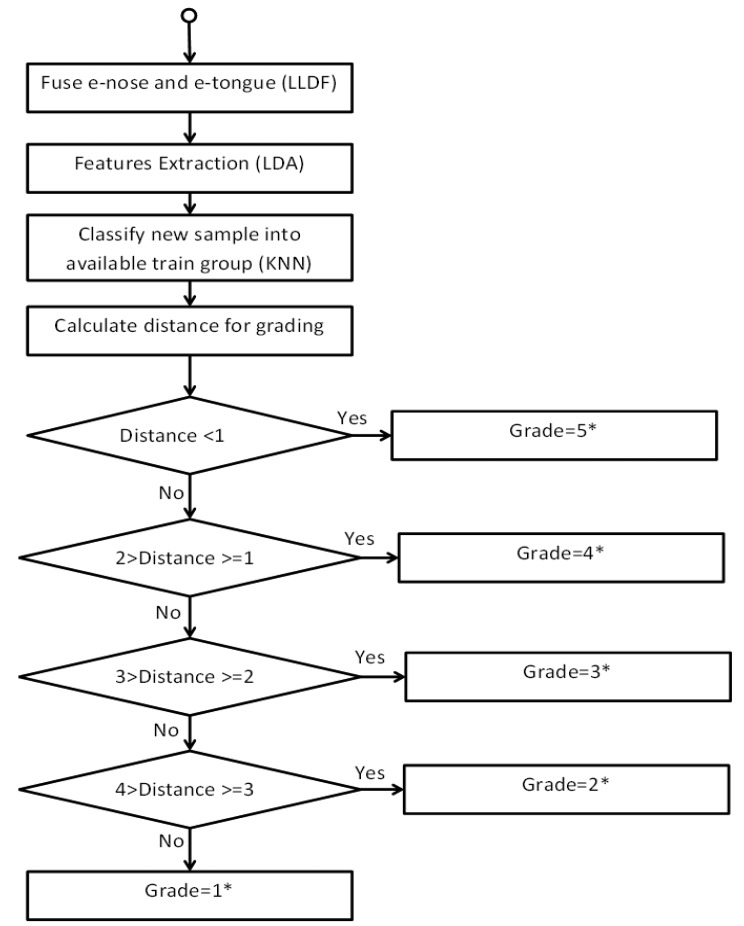
Grading algorithm.

The grading approach is illustrated in further detail and the particular algorithm applied is demonstrated in Figure 6. Flavour of the test samples is graded based on five grades that include Grade 1, 2, 3, 4, and 5 subject to different unit distance. The lowest distance corresponds to a good grade while the highest distance indicates a bad grade. To test the effectiveness of the system, we attempted to run the system with several new samples generated through simulation.

**Figure 7.**
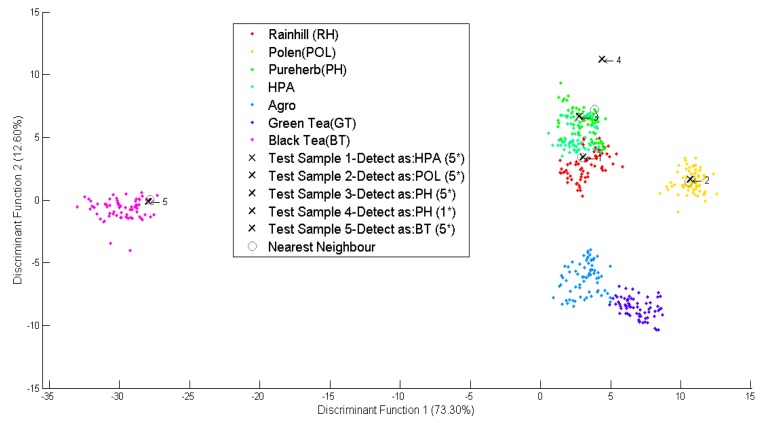
Scatter plot for different types and brands.

As shown in Figure 7, five new simulated data marked as “x”, were classified into five different groups namely RH, POL, PH, HPA and BT. The first, second, and third datasets were identified to meet the standards of the Rainhill, Polens, and Pureherb brand with the highest score of five star (5 *). The fourth data was identified to meet the Pureherb brand standard, but with one star (1 *). And the fifth data was identified as Black Tea and meets the standard for local brand with the highest five stars (5 *).

**Figure 8.**
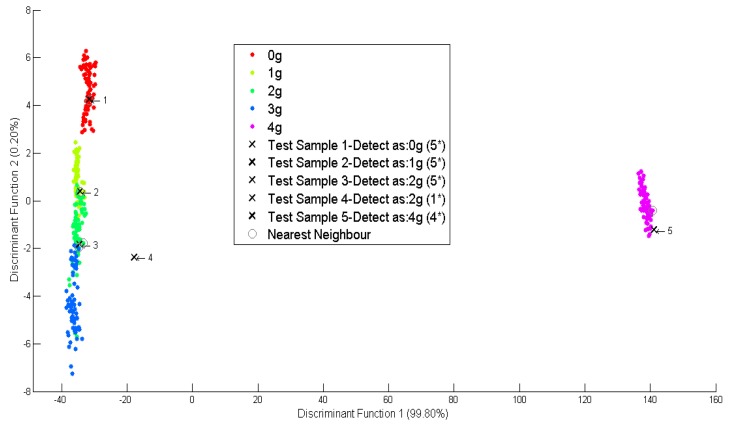
Scatter plot for different concentrations.

For different concentrations, the first simulated data is identified as not containing any *O. stamineus* as shown in Figure 8. Besides, two of the simulated data were identified having 2 g of *O. stamineus* but with different strength levels; five (5 *) and one (1 *), while the others are identified as containing 1 g and 4 g of *O. stamineus*. For the fourth dataset, the confidence to quantify the solution containing 2 g of *O. stamineus* is very low. This indicates that the amount of *O.stamineus* is closer to 3 g.

**Figure 9.**
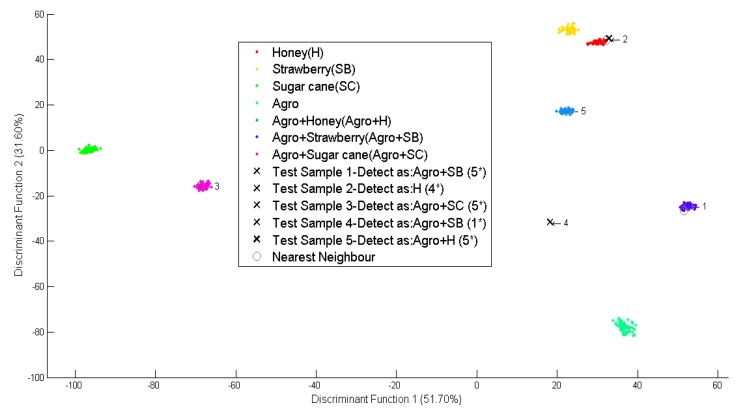
Scatter plot for different masking agents.

In Figure 9, the second simulated data point was identified as one of the masking agents (honey) with four stars (4 *) while the rest of the simulated data was identified as flavour masked *O. stamineus* with different flavours and confidence level. All data have the highest confidence level according to their respective masking agent, except the fourth simulated dataset which has the lowest level (1 *). The fourth simulated dataset had the lowest level, perhaps because the tea infusion contained small amounts of strawberry flavour or not.

## 4. Conclusions

Generally, the bio-inspired flavour assessment of herbal teas which includes three criteria; different types and brands, different concentrations, and different flavour masking agents using the two data fusion frameworks, that are LLDF and ILDF, is successful. However, flavour assessment results using GC-MS TIC data, which cover the assessment of different types and brands, as well as different concentrations were fairly low, especially for the latter criteria. Overall, for the LLDF framework, the best classifier for allocating tea samples based on different types and brands, and different concentrations is KNN, while for the flavour masking agents, PNN outperformed the rest of the classifiers. For the sensitivity and specificity point of view, KNN and LDA achieved perfect scores in the classification of tea samples based on the types and brands.

As a whole, sensitivity and specificity for the applied classifiers were good in classifying herbal teas according to different types and brand, as well as for different masking agents, but not for the different concentrations. It seems that in different concentrations, classification is harder to achieve. For the ILDF framework, again KNN turn out to be the best classifier in the classification of the three flavour masking assessments. However the pattern performance for all the classifiers from the sensitivity and specificity viewpoint generally were almost the same as in the LLDF framework. We can conclude that all the performance of all the classifiers decreases when the samples to be tested involve different concentrations. Overall, the classification findings for LLDF and ILDF were almost identical where KNN was considered the best classifier for both fusion frameworks, though the classifier ranking in ILDF for classification based on flavour masking agent was 0.4% lower that with PNN. Results from the GC-MS TIC were also different. For the classification of herbal teas based on different types and brands, the best classifier is KNN with zero error followed by SVM, LDA and PNN. Unfortunately, the classification of herbal teas using different concentrations and GC-MS TIC data was poor. The best classifiers are LDA and SVM with 60% correctly classified, while KNN scored at 55% and the worst was PNN with a 25% score.

GC-MS TIC represents the summed intensity across the entire range of masses being detected at every point in the analysis. It seems that this data provides less information to discriminate the different criteria of herbal teas. Unlike the original different criteria data, GC-MS TIC data was analysed without applying any feature selection technique. Therefore, we assumed that GC-MS TIC data is not a suitable dataset to be considered for classification analysis of herbal teas. Further analysis using features selection approach for GC-MS TIC data can be done to see whether the classification performances may be improved or remain the same. The findings of all the above analyses were further applied in the proposal of an automatic bio-inspired flavour assessment and grading system. The proposed system that has been tested using a simulated dataset shows the potential use of automation in flavour grading of herbal tea. This can be marked as an initial achievement of the bio-inspired flavour assessment technique. The application of the system may be generalized to other tea products as well as other food and beverage products but with a proper analyses procedure. It is the hope that the proposed system is able to improve the conventional methods of assessment and grading of similar products.

## Figures and Tables

**Table 1.** List of sensors description and their references in e-nose.

Sensor Number	General Description
S1	Aromatic compounds
S2	Very sensitive to nitrogen oxides
S3	Ammonia, used as sensor for aromatic compounds
S4	Mainly hydrogen, selectively (breath gases)
S5	Alkenes, aromatic compounds, less polar compounds
S6	Sensitive to methane broad range
S7	Reacts on sulphur compounds
S8	Detects alcohols, partially aromatic compounds
S9	Aromatics compounds, sulphur organic compounds
S10	Reacts on high concentrations

**Table 2.** Total observations used for training and testing stage.

Dataset	Total Number of Observation	

	Training	Testing
Different types and brands of herbal tea	490	245
Different concentrations of herbal tea	350	175
Different flavour masking agents	490	245

**Table 3.** List of spread values used for PNN classifier for different dataset.

Dataset	Spread		

	LLDF	ILDF	GC-MS
Different types and brands of herbal tea	0.079	0.079	0.001
Different concentrations of herbal tea	0.031	0.181	0.001
Different flavour masking agents	0.18	0.18	-

**Table 4.** List of parameter values set for the KNN classifier for different datasets.

Dataset	k		Distance Metrics	
	
	Fusion	GC-MS	Fusion	GC-MS
Different types and brands of herbal tea	1	2	Mahalanobis	Chebychev
Different concentrations of herbal tea	3	1	Cityblock	Euclidean
Different flavour masking agents	1	-	Cityblock	-

**Table 5.** List of parameter values set for the SVM classifier for different datasets.

Dataset	Kernel Function	Method		Box Constraint (C = 2*^N^*)		Gamma (2*^N^*)	

		Fusion	GC-MS	Fusion	GC-MS	Fusion	GC-MS
Different type of tea and brands	RBF	LS	SMO	N = 9	N = 15	N = 3	N = 10
Different concentration of herbal tea	RBF	LS	QP	N = 5	N = 7	N = 1	N = 6
Different flavour masking agent	Linear	SMO		N = 5		-	-

**Table 6.** Standardized canonical discriminant function coefficients.

**Function**						
	1	2	3	4	5	6
*EN*1	2.240	4.446	0.393	2.010	−2.407	1.735
*EN*2	−1.153	0.336	−0.428	−0.233	−0.035	0.334
*EN*3	2.823	−7.290	1.911	−2.626	3.175	−0.183
*EN*4	0.580	−0.712	1.499	0.252	1.191	0.616
*EN*5	−3.991	2.763	−2.016	1.082	0.189	−0.866
*EN*6	−0.884	0.305	1.383	1.238	0.211	−1.398
*EN*7	−1.539	−1.427	−1.014	2.754	−1.663	−0.884
*EN*8	−0.327	−1.569	−2.309	−1.249	−1.577	1.844
*EN*9	2.915	1.024	1.460	−2.287	3.095	1.322
*EN*10	1.797	2.620	0.511	−0.116	0.439	−0.813

**Table 7.** Summary of the total number of useful discriminant scores.

Dataset	No. of Groups(*G*)	No. of Features(*p*)	No. of Discriminant Function and Score = min (*G* – 1, *p*)
Different concentrations of herbal tea	5	10	4
Different flavour masking agents	7	10	6
Different types and brands of herbal tea	7	10	6

**Table 8.** Standardized canonical discriminant function coefficients.

**Function**						
	1	2	3	4	5	6
*ET*1	5.657	4.446	0.393	2.010	−2.407	1.735
*ET*2	2.063	0.336	−0.428	−0.233	−0.035	0.334
*ET*3	−0.182	−7.290	1.911	−2.626	3.175	−0.183
*ET*4	0.165	−0.712	1.499	0.252	1.191	0.616
*ET*6	−0.099	2.763	−2.016	1.082	0.189	−0.866
*ET*7	−0.478	0.305	1.383	1.238	0.211	−1.398
*ET*8	0.160	−1.427	−1.014	2.754	−1.663	−0.884
*ET*9	−4.656	−1.569	−2.309	−1.249	−1.577	1.844
*ET*11	−0.113	1.024	1.460	−2.287	3.095	1.322

**Table 9.** Summary of the total number of useful discriminant scores.

Dataset	No. of Groups (*G*)	No. of Features (*p*)	No. of Discriminant Function and Score = min (*G*–1, *p*)	Unresponsive Features
Different concentrations of herbal tea	5	11	4	-
Different flavour masking agents	7	11	6	-
Different types and brands of herbal tea	7	11	6	*ET*5 and *ET*10

**Table 10.** Comparison of different type of classifiers' PPV for different types and brands dataset in LLDF framework (in percentages).

		RH	POL	PH	HPA	Agro	GT	BT	Accuracy
**Training**	LDA	100.0	100.0	100.0	100.0	100.0	100.0	100.0	100.0
	KNN	100.0	100.0	100.0	100.0	100.0	100.0	100.0	100.0
	SVM	100.0	100.0	100.0	100.0	100.0	100.0	100.0	100.0
	PNN	100.0	100.0	85.7	95.7	100.0	100.0	100.0	97.3

**Testing**	LDA	100.0	100.0	100.0	100.0	100.0	100.0	100.0	100.0
	KNN	100.0	100.0	100.0	100.0	100.0	100.0	100.0	100.0
	SVM	100.0	100.0	100.0	100.0	74.3	100.0	100.0	96.3
	PNN	100.0	100.0	85.7	94.3	100.0	100.0	100.0	97.1

**Table 11.** Comparison of different classifiers' sensitivity and specificity for different types and brands dataset in LLDF framework (in percentages).

		RH	POL	PH	HPA	Agro	GT	BT
**Sensitivity**	LDA	100.0	100.0	100.0	100.0	100.0	100.0	100.0
	KNN	100.0	100.0	100.0	100.0	100.0	100.0	100.0
	SVM	100.0	83.3	100.0	100.0	100.0	100.0	94.6
	PNN	92.1	100.0	93.8	94.3	100.0	100.0	100.0

**Specificity**	LDA	100.0	100.0	100.0	100.0	100.0	100.0	100.0
	KNN	100.0	100.0	100.0	100.0	100.0	100.0	100.0
	SVM	100.0	100.0	100.0	100.0	95.9	100.0	100.0
	PNN	100.0	100.0	97.7	99.0	100.0	100.0	100.0

**Table 12.** Comparison of different types of classifiers' PPV for different concentrations (Agro) dataset in LLDF framework (in percentages).

		Distilled Water	1 g	2 g	3 g	4 g	Accuracy
**Training**	LDA	97.1	91.4	82.9	85.7	100.0	91.4
	KNN	100	100	100	100	100	100
	SVM	100	100	97	100	100	99
	PNN	100	100	100	100	100	100

**Testing**	LDA	97.1	62.9	71.4	91.4	100.0	84.6
	KNN	100.0	100.0	82.9	94.3	100.0	95.4
	SVM	100.0	71.4	85.7	80.0	100.0	87.4
	PNN	100.0	91.4	68.6	88.6	100.0	89.7

**Table 13.** Comparison of different type of classifiers' sensitivity and specificity for different concentrations (Agro) dataset in LLDF framework (in percentages).

		Distilled Water	1 g	2 g	3 g	4 g
**Sensitivity**	LDA	100.0	87.2	88.0	78.1	72.7
	KNN	100.0	97.2	93.5	86.8	100.0
	SVM	77.8	100.0	100.0	100.0	74.5
	PNN	100.0	86.5	82.8	79.5	100.0

**Specificity**	LDA	100.0	99.0	88.7	93.0	96.9
	KNN	100.0	100.0	95.8	98.5	100.0
	SVM	100.0	93.3	96.6	95.2	100.0
	PNN	100.0	97.8	92.5	97.1	100.0

**Table 14.** Comparison of different type of classifiers' PPV for each class of different types of masking agent dataset in LLDF framework (in percentages).

		H	SB	SC	Agro	Agro + H	Agro + SB	Agro + SC	Accuracy
**Training**	LDA	100.0	100.0	100.0	100.0	100.0	100.0	100.0	100.0
	KNN	100.0	100.0	100.0	100.0	100.0	100.0	100.0	100.0
	SVM	100.0	100.0	100.0	100.0	100.0	100.0	100.0	100.0
	PNN	100.0	100.0	100.0	100.0	100.0	100.0	100.0	100.0

**Testing**	LDA	100.0	100.0	100.0	97.1	100.0	100.0	100.0	99.6
	KNN	100.0	100.0	100.0	97.1	100.0	100.0	100.0	99.6
	SVM	100.0	100.0	100.0	94.3	100.0	100.0	100.0	99.2
	PNN	100.0	100.0	100.0	100.0	100.0	100.0	100.0	100.0

**Table 15.** Comparison of different type of classifiers' sensitivity and specificity for each class of different types of masking agent dataset in LLDF framework (in percentages).

		H	SB	SC	Agro	Agro + H	Agro + SB	Agro + SC
**Sensitivity**	LDA	100.0	100.0	100.0	100.0	100.0	97.2	100.0
	KNN	100.0	100.0	100.0	100.0	100.0	97.2	100.0
	SVM	100.0	100.0	100.0	100.0	100.0	100.0	94.6
	PNN	100.0	100.0	100.0	100.0	100.0	100.0	100.0

**Specificity**	LDA	100.0	100.0	100.0	99.5	100.0	100.0	100.0
	KNN	100.0	100.0	100.0	99.5	100.0	100.0	100.0
	SVM	100.0	100.0	100.0	99.1	100.0	100.0	100.0
	PNN	100.0	100.0	100.0	100.0	100.0	100.0	100.0

**Table 16.** Comparison of different type of classifiers' PPV for each class in different types and brands dataset in ILDF framework (in percentages).

		RH	POL	PH	HPA	Agro	GT	BT	Accuracy
**Training**	LDA	100.0	100.0	100.0	100.0	100.0	100.0	100.0	100.0
	KNN	100.0	100.0	100.0	100.0	100.0	100.0	100.0	100.0
	SVM	100.0	100.0	100.0	100.0	100.0	100.0	100.0	100.0
	PNN	100.0	100.0	100.0	100.0	100.0	100.0	100.0	100.0

**Testing**	LDA	100.0	100.0	97.1	100.0	100.0	100.0	100.0	99.6
	KNN	100.0	100.0	100.0	100.0	100.0	100.0	100.0	100.0
	SVM	100.0	100.0	100.0	100.0	71.4	100.0	100.0	95.9
	PNN	100.0	100.0	100.0	100.0	71.4	100.0	100.0	95.9

**Table 17.** Comparison of different type of classifiers' sensitivity and specificity for each class of different types and brands dataset in ILDF framework (in percentages).

		RH	POL	PH	HPA	Agro	GT	BT
**Sensitivity**	LDA	100.0	100.0	100.0	97.2	100.0	100.0	100.0
	KNN	100.0	100.0	100.0	100.0	100.0	100.0	100.0
	SVM	100.0	100.0	87.5	100.0	100.0	100.0	87.5
	PNN	50.0	100.0	100.0	100.0	100.0	100.0	100.0

**Specificity**	LDA	100.0	100.0	99.5	100.0	100.0	100.0	100.0
	KNN	100.0	100.0	100.0	100.0	100.0	100.0	100.0
	SVM	100.0	100.0	100.0	100.0	95.5	100.0	100.0
	PNN	100.0	99.5	99.1	98.6	92.9	98.1	95.9

**Table 18.** Comparison of different type of classifiers' PPV for each class of different concentrations dataset in ILDF framework (in percentages).

		Distilled Water	1 g	2 g	3 g	4 g	Accuracy
**Training**	LDA	97.1	92.9	81.4	85.7	100.0	91.4
	KNN	100.0	100.0	100.0	100.0	100.0	100.0
	SVM	100.0	100.0	100.0	98.6	100.0	99.7
	PNN	100.0	100.0	100.0	100.0	100.0	100.0

**Testing**	LDA	97.1	57.1	74.3	88.6	100.0	83.4
	KNN	100.0	80.0	91.4	100.0	100.0	94.3
	SVM	85.7	71.4	85.7	74.3	100.0	83.4
	PNN	100.0	71.4	85.7	80.0	100.0	87.4

**Table 19.** Comparison of different classifier's sensitivity and specificity for each class of different concentrations dataset in ILDF framework (in percentages).

		Distilled Water	1 g	2 g	3 g	4 g
**Sensitivity**	LDA	100.0	79.1	90.9	74.3	77.5
	KNN	100.0	100.0	100.0	77.8	100.0
	SVM	100.0	100.0	93.8	100.0	56.5
	PNN	61.4	100.0	100.0	100.0	100.0

**Specificity**	LDA	100.0	94.9	97.9	94.9	100.0
	KNN	100.0	95.2	97.9	100.0	100.0
	SVM	96.6	93.3	96.5	94.0	100.0
	PNN	100.0	93.3	96.6	95.2	100.0

**Table 20.** Comparison of different type of classifiers' PPV for each class of different types of masking agent dataset in ILDF framework (in percentages).

		H	SB	SC	Agro	Agro + H	Agro + SB	Agro + SC	Accuracy
**Training**	LDA	100.0	100.0	100.0	100.0	100.0	100.0	100.0	100.0
	KNN	100.0	100.0	100.0	100.0	100.0	100.0	100.0	100.0
	SVM	100.0	100.0	100.0	100.0	100.0	100.0	100.0	100.0
	PNN	100.0	100.0	100.0	100.0	100.0	100.0	100.0	100.0

**Testing**	LDA	100.0	100.0	100.0	97.1	100.0	100.0	100.0	99.6
	KNN	100.0	100.0	100.0	100.0	100.0	100.0	100.0	100.0
	SVM	100.0	100.0	100.0	100.0	100.0	100.0	100.0	100.0
	PNN	100.0	100.0	85.7	37.1	100.0	100.0	100.0	89.0

**Table 21.** Comparison of different type of classifiers' sensitivity and specificity for each class of different types of masking agent dataset in ILDF framework (in percentages).

		H	SB	SC	Agro	Agro + H	Agro + SB	Agro + SC
**Sensitivity**	LDA	100.0	100.0	100.0	100.0	100.0	97.2	100.0
	KNN	100.0	100.0	100.0	100.0	100.0	100.0	100.0
	SVM	100.0	100.0	100.0	100.0	100.0	100.0	100.0
	PNN	56.5	100.0	100.0	100.0	100.0	100.0	100.0

**Specificity**	LDA	100.0	100.0	100.0	99.5	100.0	100.0	100.0
	KNN	100.0	100.0	100.0	100.0	100.0	100.0	100.0
	SVM	100.0	100.0	100.0	100.0	100.0	100.0	100.0
	PNN	100.0	100.0	97.7	90.5	100.0	100.0	100.0

**Table 22.** Comparison of different type of classifiers' PPV for each class using GC-MS of different types and brands dataset (in percentages).

		RH	POL	PH	HPA	Agro	GT	BT	Accuracy
**Training**	LDA	100.0	100.0	100.0	100.0	100.0	100.0	100.0	100.0
	KNN	100.0	100.0	100.0	100.0	100.0	100.0	100.0	100.0
	SVM	100.0	100.0	100.0	100.0	100.0	100.0	100.0	100.0
	PNN	100.0	100.0	100.0	100.0	100.0	100.0	100.0	100.0

**Testing**	LDA	100.0	100.0	80.0	100.0	100.0	100.0	80.0	94.3
	KNN	100.0	100.0	100.0	100.0	100.0	100.0	100.0	100.0
	SVM	100.0	100.0	100.0	100.0	100.0	100.0	80.0	97.1
	PNN	100.0	100.0	0.0	100.0	100.0	100.0	0.0	71.4

**Table 23.** Comparison of different type of classifiers' sensitivity and specificity for each class of different types and brands dataset using GC-MS (in percentages).

		RH	POL	PH	HPA	Agro	GT	BT
**Sensitivity**	LDA	100.0	83.3	100.0	100.0	100.0	83.3	100.0
	KNN	100.0	100.0	100.0	100.0	100.0	83.3	100.0
	SVM	100.0	100.0	100.0	100.0	83.3	100.0	100.0
	PNN	33.3	100.0	0.0	100.0	100.0	100.0	0.0

**Specificity**	LDA	100.0	100.0	96.8	100.0	100.0	100.0	96.8
	KNN	100.0	100.0	100.0	100.0	100.0	100.0	96.8
	SVM	100.0	100.0	100.0	100.0	100.0	100.0	96.8
	PNN	100.0	100.0	85.7	100.0	100.0	100.0	85.7

**Table 24.** Comparison of different type of classifiers' PPV for each class of different concentrations (Agro) dataset using GC-MS (in percentages).

		1 g	2 g	3 g	4 g	Accuracy
**Training**	LDA	100.0	100.0	100.0	100.0	100.0
	KNN	100.0	100.0	100.0	100.0	100.0
	SVM	100.0	100.0	100.0	100.0	100.0
	PNN	100.0	100.0	100.0	100.0	100.0

**Testing**	LDA	40.0	80.0	80.0	40.0	60.0
	KNN	60.0	40.0	80.0	40.0	55.0
	SVM	80.0	40.0	80.0	40.0	60.0
	PNN	100.0	0.0	0.0	0.0	25.0

**Table 25.** Comparison of different type of classifiers' sensitivity and specificity for each class of different concentrations dataset using GC-MS (in percentages).

		1 g	2 g	3 g	4 g
**Sensitivity**	LDA	66.7	50.0	57.1	100.0
	KNN	42.9	50.0	57.1	100.0
	SVM	80.0	50.0	57.1	50.0
	PNN	25.0	0.0	0.0	0.0

**Specificity**	LDA	82.4	91.7	92.3	83.3
	KNN	84.6	81.3	92.3	83.3
	SVM	93.3	81.3	92.3	81.3
	PNN	0.0	75.0	75.0	75.0
